# Research on Storage Grain Temperature Prediction Method Based on FTA-CNN-SE-LSTM with Dual-Domain Data Augmentation and Deep Learning

**DOI:** 10.3390/foods14101671

**Published:** 2025-05-09

**Authors:** Hailong Peng, Yuhua Zhu, Zhihui Li

**Affiliations:** 1 College of Information Science and Engineering, Henan University of Technology, Zhengzhou 450001, China; hlongpeng@163.com (H.P.); 18961863293@163.com (Y.Z.); 2 Key Laboratory of Grain Information Processing and Control, Ministry of Education, Henan University of Technology, Zhengzhou 450001, China

**Keywords:** data augmentation, grain storage temperature prediction, Gaussian noise, fast Fourier transform

## Abstract

Temperature plays a crucial role in the grain storage process and food security. Due to limitations in grain storage data acquisition in real-world scenarios, this paper proposes a data augmentation method for grain storage data that operates in both the time and frequency domains, as well as an enhanced grain storage temperature prediction model. To address the issue of small sample sizes in grain storage temperature data, Gaussian noise is added to the grain storage temperature data in the time domain to highlight the subtle variations in the original data. The fast Fourier transform (FFT) is employed in the frequency domain to highlight periodicity and trends in the grain storage temperature data. The prediction model uses a long short-term memory (LSTM) network, enhanced with convolution layers for feature extraction and a Squeeze-and-Excitation Networks (SENet) module to suppress unimportant features and highlight important ones. Experimental results show that the FTA-CNN-SE-LSTM compares with the original LSTM network, and the MAE and RMSE are reduced by 74.77% and 74.02%, respectively. It solves the problem of data limitation in the actual grain storage process, greatly improves the accuracy of grain storage temperature prediction, and can accurately prevent problems caused by abnormal grain pile temperature.

## 1. Introduction

During the long grain storage period, grain piles are influenced by various factors, including temperature, humidity, pests, gas, and mold. However, temperature exerts the most significant impact on the quality of stored grain [1]. Once the temperature of a grain pile exceeds the critical temperature for safe grain storage, the grain pile becomes susceptible to mold, pests, condensation, and other issues [2], which will lead to grain loss. Therefore, in order to improve the quality of stored grain and ensure food safety, it is crucial to establish a model capable of accurately predicting temperature changes of grain piles.

Traditional grain storage temperature prediction [3] first requires obtaining a large amount of data on grain storage conditions, then using mathematical, physical, and statistical methods to establish the mathematical relationship between various factors and grain pile temperature based on large-scale analysis of the data, and finally completing the construction of the grain temperature prediction model. This means that the traditional model will have high complexity and a large amount of data calculation. Additionally, the geographical location and type of granaries reflect the non-uniqueness of the physical conditions of grain storage [4]. The traditional grain storage temperature prediction model will be difficult to reflect universality with. With the development of grain information technology, machine learning and deep learning technologies have been widely employed in grain storage temperature prediction [5], effectively addressing the challenges of low prediction accuracy, complex structures, and a large number of parameters in traditional models. Due to its powerful multi-layer nonlinear transformation capabilities and feature extraction and generalization capabilities, the grain storage temperature prediction model based on machine learning and deep learning offers greater universality [6].

In recent years, scholars have applied machine learning and deep learning technologies to the prediction of stored grain temperature [7]. Zhang Yinhua et al. [8] improved the nonlinear intelligent modeling method of the RBF neural network based on the genetic algorithm of the cloud model, applied the cloud genetic RBF neural network algorithm to the prediction of grain pile moisture and heat, and accurately predicted the trend of grain pile moisture and heat change. Guo Pingfei et al. [9] proposed the GANPSO-BP network for grain temperature prediction based on the BP neural network and borrowed the ideas of the genetic algorithm and particle swarm algorithm. This approach significantly improved the algorithm’s performance compared to the basic network and effectively predicted grain temperature changes. Xie Hui et al. [10] integrated the genetic algorithm into the GRU model, added a dropout layer, and optimized model parameters through several experiments to construct the GA-GRU grain temperature prediction model. Huang Qilan et al. [11] proposed a PSO-LSSVM grain condition prediction model based on particle swarm algorithm and the least squares support vector machine. Zhang Xiao et al. [12] constructed a grain pile temperature prediction model based on the ARMA model and SVR technology, used a time series analysis model to fit the linear part of the prediction result, introduced a residual factor, and employed a support vector regression model to fit the nonlinear part. Hou Jia et al. [13] used the gray wolf algorithm to optimize the parameters of the support vector regression model based on the SVR prediction model and established a GWO-SVR hybrid grain condition prediction model. Liu Kaifei et al. [14] proposed a grain temperature prediction model based on GRU. In order to reduce the complexity of the model and improve the prediction accuracy, the PSO and GA algorithms were used to optimize the model parameters, and the attention mechanism layer was introduced. The dropout method was used to solve the problem of model overfitting, and the Adam optimization algorithm was used to solve the problem of the model falling into the local optimal point due to the fixed learning rate.

Most of the research work on grain storage temperature prediction mentioned above is limited to the model optimization level, without considering the optimization problem from the perspective of grain storage data. First of all, starting from data acquisition, various grain storage data, including temperature, humidity, etc., need to be collected through numerous sensor devices [15]. Ideally, to facilitate the quality of the subsequent prediction model training, continuous, long-term data collection is necessary to ensure the acquisition of a large volume of high-quality data. However, in reality, most grain storage data in grain depots still rely on manually activated sensors, with uncertainty in the data collection times. This indirectly leads to the lack of grain storage data and makes it difficult to meet the requirements for model training. As for the grain storage data itself, grain temperature is not a single factor. It will be affected by various factors such as air temperature, grain warehouse humidity, and air humidity, and there are also mutual influences between factors [16]. As a form of typical time series data [17], grain temperature has a series of time-generated characteristics such as trend, periodicity, volatility, stability, and symmetry, which lead to signification distribution changes over time.

To this end, this paper proposes a method for predicting stored grain temperature based on time domain and frequency domain augmentation, combined with deep learning, from a data perspective to overcome the above problems. The contributions of this method are as follows:

(1) From the data perspective, Gaussian noise is added to augment the data in the time domain, while fast Fourier transform is used to expand the grain storage data into the frequency domain for further augmentation. The dual-domain augmentation of grain storage data, both in time and frequency, improves the utilization value of scarce data and makes a great contribution to the performance improvement of the grain storage temperature prediction model in the subsequent step.
(2) The CNN-LSTM network model is constructed by combining convolutional neural networks and long short-term memory networks, giving full play to the respective advantages of convolutional neural networks in extracting data features and LSTM networks in time series feature mining and time series modeling.
(3) The SENet (Squeeze-and-Excitation Networks) module is introduced to improve the channel extraction of local features, increase the selection of key information, suppress unimportant features, and reduce the number of model parameters.

## 2. Materials and Methods

### 2.1. Acquisition of Grain Storage Temperature Data

**Figure 1:**
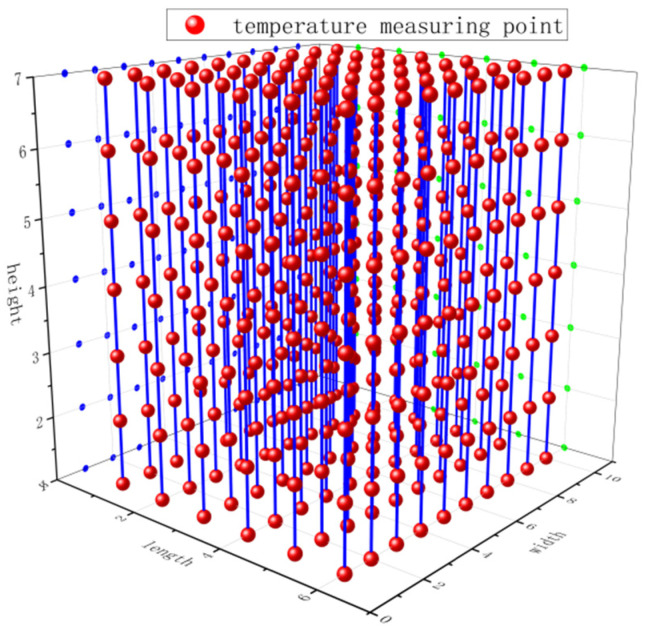
Layout of temperature measurement cables in experimental granary.

In order to obtain the real temperature data of stored grain, temperature-measuring cables were arranged in a bungalow of a grain warehouse in Yushu City, Jilin Province. The bungalow is 36 m long, 24 m wide, and 6 m high with grain stored. The stored grain is corn. To fully consider the grain temperature at each point in the granary, the temperature measuring cables were arranged according to national standards. Each temperature measuring point was 5 m apart horizontally, 1.8 m apart vertically, and 0.5 m apart from the grain surface, the ground, and the warehouse wall. According to this standard, the granary was planned as 6 rows × 10 columns × 7 layers, with a total of 420 temperature measuring points, and the temperature measuring cables were arranged according to the planned positions. Grain temperature was measured every four hours. The layout of the temperature measuring cables is shown in Figure 1.

### 2.2. Data Augmentation

As a strategic storage resource for the country, the data acquisition of grain has certain limitations. Additionally, due to the current status of grain storage monitoring and control, data acquisition relies on a lot of time, manpower, and hardware costs, resulting in a limited amount of grain storage data for model input. This indirectly impacts the prediction performance of the model. The introduction of data augmentation technology will enable the model to fully utilize the limited amount of grain storage data and learn the underlying patterns of grain storage dynamics. As the most typical form of time series data, grain storage data itself are augmented in both the time domain and frequency domain. The augmented new data samples can have higher diversity while retaining the periodicity and trend characteristics of the original data.

#### 2.2.1. Time Domain Augmentation

The grain storage temperature data are a collection of data arranged in chronological order in the time dimension, which has a high dependence on time. This directly determines that all the characteristics of the data will be generated around time, such as seasonality, periodicity, trend, etc. However, due to the limited amount of grain storage data itself, it is challenging to achieve the ideal level of accuracy of the prediction model by learning only through the characteristics of the data itself. Therefore, in the time domain dimension, the grain storage temperature data are augmented by adding Gaussian noise.

**Figure 2:**
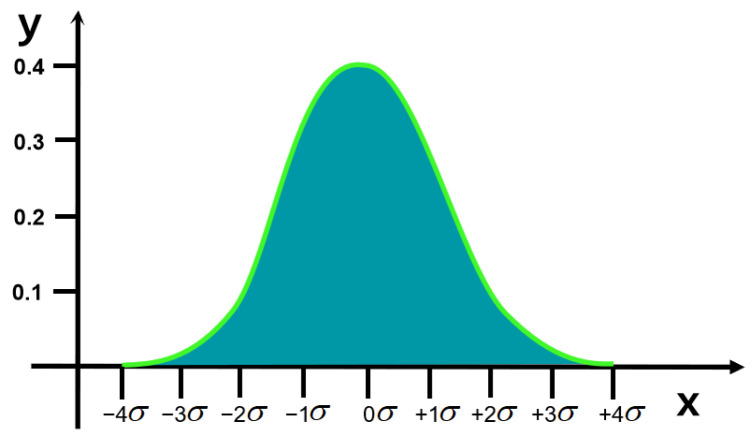
Gaussian distribution.

In mathematics, Gaussian noise [18] is a type of noise generated by adding Gaussian distributed random values with a mean of 0 and a standard deviation of σ to the input data. Gaussian distribution, also known as normal distribution, is a continuous probability distribution, as illustrated in Figure 2.

(1) $$f(x;\mu ,\sigma )=\frac{1}{\sqrt{2\pi \sigma^{2}}}e^{-\frac{{\left(x-\mu \right)}^{2}}{2\sigma^{2}}}$$

where x is a random variable, μ is the mean, and σ is the standard deviation.

In the field of deep learning, one of the most common uses of Gaussian noise is to be added to input data. In computer vision, Gaussian noise can be added to each image before training, allowing the model to learn small variations in the input image data, thereby improving its robustness. The introduction of Gaussian noise can also replace stains and blurred areas in the image, so even if the image to be detected in real applications is different from the training image, it can still be efficiently and accurately identified, effectively avoiding overfitting.

In the field of time series prediction, we can also draw on computer vision data augmentation technology and introduce the concept of Gaussian noise [19]. Before training the prediction model, random values with a mean of zero and a normal distribution with a given standard deviation can be added to the input grain storage temperature data to generate noise. This makes some small changes in the grain storage temperature data more robust and better highlights the hard-to-find data. This indirectly enables the prediction model to pay more attention to small temperature characteristics and changes during the training and learning process with a limited data set, thereby improving the accuracy and robustness of the model prediction.

#### 2.2.2. Frequency Domain Augmentation

In the frequency domain, grain storage temperature data can also be augmented. Frequency domain augmentation expands the grain storage temperature data from the time domain to the frequency domain, decomposes the temperature time series data into different frequency components, and expands the data to a certain extent, which helps to analyze and process the periodicity and trend of the data beyond the time domain. From the perspective of data characteristics, grain storage temperature data are similar to data such as residential electricity consumption, traffic flow, and commodity sales. This form of data is affected by external factors and exhibits high periodicity. This periodic data can be easily decoupled in the frequency domain, and corresponding operations can be performed.

The fast Fourier transform (FFT) was proposed by American engineers Cooley and Tukey in 1965 [20]. It is one of the most commonly used augmentation techniques in the field of frequency domain augmentation. It is actually based on the discrete Fourier transform (DFT) and decomposes the long-sequence DFT into the sum of many short-sequence DFTs according to the periodicity and symmetry in the DFT [21]. The DFT converts the discrete time series into the frequency domain and decomposes a time series of finite length into a combination of sine waves and cosine waves, which can then analyze the frequency components of the data signal and help capture the periodicity of the time series data. The given sequence is defined as $X=\{X_{n}\}$, the length is defined as N, $X_{k}$ is the relative amplitude of discrete frequency points corresponding to $X_{n}$, k is the frequency index, and $n\in \left[0,n-1\right]$, and then the definition of DFT is given as

(2) $$X_{k}=\sum_{n=0}^{N-1}x_{n}e^{-\frac{2\pi i}{N}nk},0\leq k\leq N-1$$

The rotation $W_{N}=e^{-\frac{2\pi }{N}nk}i$ factor is defined as the imaginary symbol, and the FFT transform is defined as

(3) $$X(k)=\mathrm{DFT}[x(n)]=\sum_{n=0}^{N-1}x(n)W_{N}^{kn},\;k=0,1,\cdots ,N-1$$

If the amount of DFT calculation is proportional to $N^{2}$, then for long sequences, to reduce the computational load, the long-sequence DFT can be decomposed into the sum of several shorter-sequence DFTs. By mapping the complex array index obtained by the FFT operation to the corresponding frequency, the phase and amplitude of each frequency component are obtained. $f_{k}$ is defined as the driver frequency of the frequency component, $f_{T}$ is the sampling frequency, and s is the array index of the FFT operation result. The expression between frequency and index is defined as follows:

(4) $$f_{k}=\frac{k_{s}f_{T}}{N}$$

**Figure 3:**
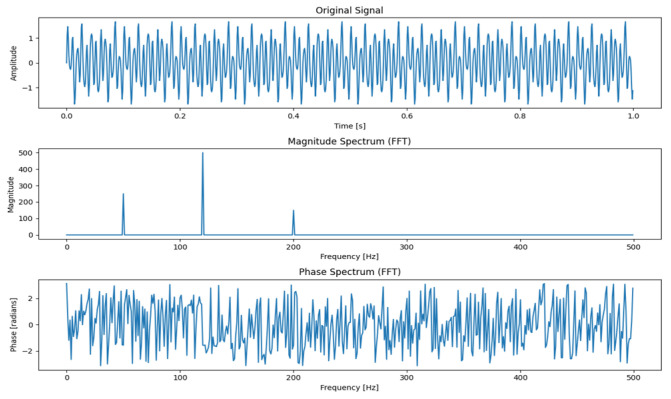
Fast Fourier transform effect.

By analyzing the different frequency intensities through spectrum analysis, the main frequency components are obtained, and the periods of each sequence are calculated. The effect of FFT is shown in Figure 3.

#### 2.2.3. Augmentation of Grain Storage Temperature Data

**Figure 4:**
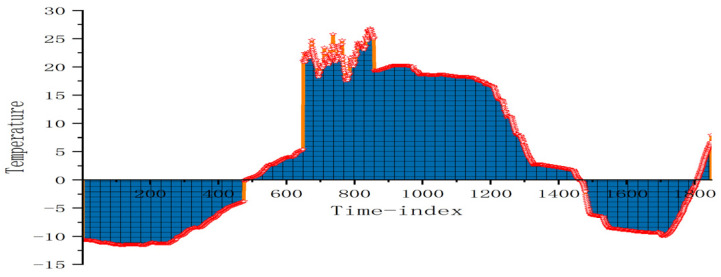
Distribution of original grain storage temperature data.

Due to the limited amount of grain storage temperature data and the data quality and quantity issues caused by sensor equipment, Gaussian noise and FFT are introduced to enhance the data in both the time domain and frequency domain dimensions. In the experiment, the distribution of grain storage temperature data obtained by the arranged temperature measurement cables is shown in Figure 4. It can be observed that some data segments have large gaps, which are caused by human or sensor equipment during the measurement process. The amount of data is also small, which will lead to poor model training results. The dual-domain augmentation module of time domain and frequency domain proposed in this paper can effectively address this problem.

**Figure 5:**
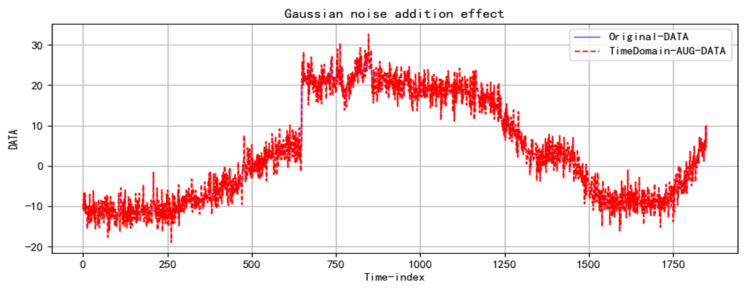
Distribution of grain storage temperature data with Gaussian noise added.

The Gaussian noise intensity (i.e., the standard deviation) is set to 0.01, with the mean fixed at 0. These parameter settings account for the stable variation in grain storage temperature data, simulating small random errors in actual measurements without significantly affecting the data’s core characteristics, thus effectively expanding the dataset. The data with added Gaussian noise are shown in Figure 5. This approach reduces the gaps between data segments caused by measurement and control issues, while also highlighting minor changes in the grain storage temperature’s development process. As a result, the prediction model can better capture the data’s characteristics and fluctuations, improving both accuracy and robustness.

**Figure 6:**
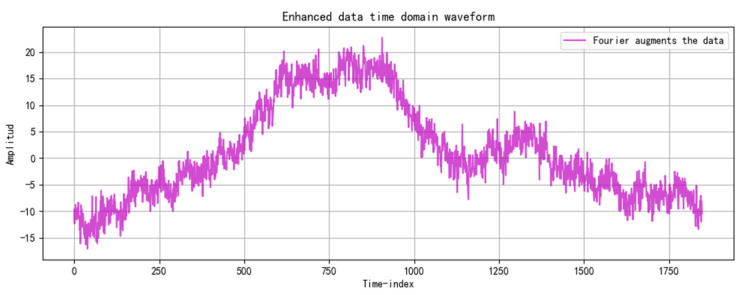
Distribution of stored grain temperature data after fast Fourier transform.

**Figure 7:**
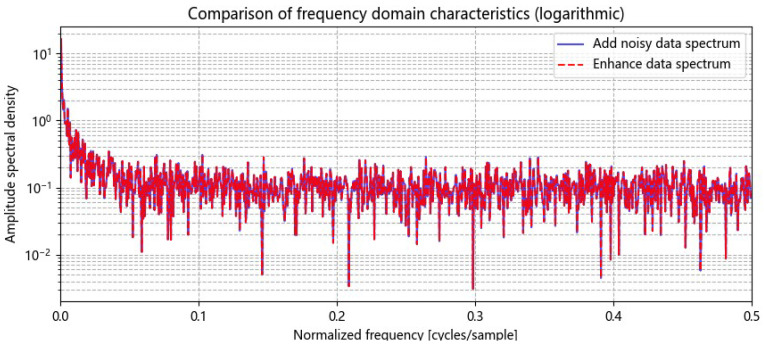
Comparison of the frequency domain characteristics of stored grain temperature data before and after fast Fourier transform.

**Figure 8:**
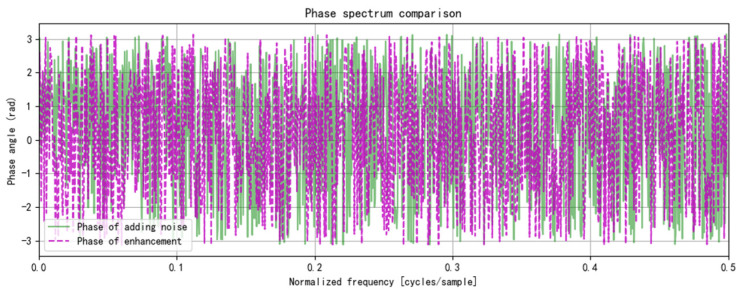
Comparison of phase spectra of stored grain temperature data before and after fast Fourier transform.

To enhance the utilization of limited grain storage data, Gaussian noise is added, and the data undergo a fast Fourier transform, transitioning from the time domain to the frequency domain. A spectrum amplification factor of 1.2 is applied to emphasize the data’s characteristics in the frequency domain. This parameter is selected based on an analysis of the grain storage temperature data characteristics and prior experimental results. Increasing this factor appropriately highlights specific frequency components, aiding in the analysis of data periodicity and trends. However, care must be taken to avoid data distortion due to over-enhancement. This method decouples the periodicity and trends of grain storage temperature in the frequency domain, aiding the prediction model’s understanding of its characteristics. Combined with Gaussian noise, it enables dual-domain data enhancement in both the time and frequency domains. Figure 6 shows the fast Fourier transform results of the grain storage data with Gaussian noise, Figure 7 presents a comparison of the frequency domain characteristics before and after enhancement, and Figure 8 illustrates the phase spectrum comparison.

### 2.3. Model Proposal

#### 2.3.1. Long Short-Term Memory Neural Network

Traditional recurrent neural networks (RNNs) face the challenges of gradient vanishing and gradient exploding when processing time series data. These issues make it difficult for RNNs to capture the time dependency in time series data. To address the above problems, Hochreiter et al. [22] proposed a special recurrent neural network, the long short-term memory (LSTM) neural network, which introduced a gating mechanism and memory units to ensure effective learning and preserve time-dependent information, which is suitable for regression tasks of time series data such as grain storage temperature.

**Figure 9:**
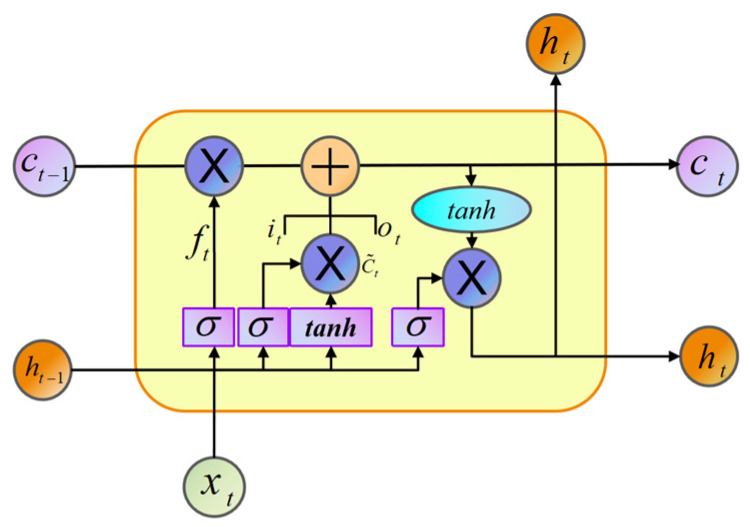
LSTM model unit structure.

LSTM [23] introduces three gates: the input gate, the forget gate, and the output gate. The input data of the input gate consist of the current grain storage temperature data and the output data of the previous unit. The forget gate is responsible for deciding which information to discard from the memory unit. The output gate uses the sigmoid activation function to decide which values in the memory unit need to be output and multiplies this value with the tanh value to obtain the information to be output. In addition, LSTM also contains hidden states and memory cells with the same form as the hidden states, thereby recording additional information in the time series data. The hidden state represents the output of LSTM at each time step and contains the information of the current time step. The memory cell is the core of LSTM and retains the grain storage temperature information from the time series data. Through the interaction between the forget gate and the input gate, it learns how to selectively remember or forget information. The model structure is shown in Figure 9.

The input of LSTM is the current time step input $X_{t}$ and the previous time step hidden state $h_{t-1}$. Assume that the number of hidden units is h, the number of samples is n, and the number of inputs is d. Given a small batch input $X_{t}$ at time step t, the input gate $i_{t}$, forget gate $f_{t}$, and output gate $o_{t}$, ${\tilde{C}}_{t}$ at time step t are expressed as sigmoid activation functions, where $W_{ix}$, $W_{fx}$, $W_{ox}$ and $W_{ih}$, $W_{fh}$, $W_{ox}$ are weight parameters, $b_{i}$, $b_{f}$, $b_{o}$ are bias parameters, the hidden state is $h_{t}$, the memory cell is $C_{t}$, and ${\tilde{C}}_{t}$ is a candidate memory cell. The LSTM unit weight update formula is as follows:

(5) $$C_{t}=\tanh(W_{cx}X_{t}+W_{ch}h_{t-1}+b_{c})$$

(6) $$i_{t}=\sigma (W_{ix}x_{t}+W_{ih}h_{t-1}+b_{i})$$

(7) $$f_{t}=\sigma (W_{fx}x_{t}+W_{fh}h_{t-1}+b_{f})$$

(8) $${\tilde{C}}_{t}=f_{t}⊗c_{t-1}+i_{t}⊗C_{t}$$

(9) $$o_{t}=\sigma (W_{ox}x_{t}+W_{oh}h_{t-1}+b_{o})$$

(10) $$h_{t}=o_{t}⊗\tanh(c_{t})$$

#### 2.3.2. CNN

**Figure 10:**
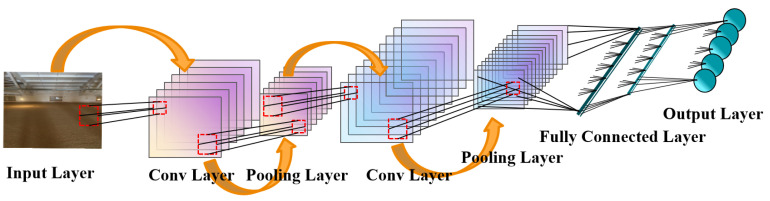
Convolutional neural network (CNN) model structure.

Convolutional neural network (CNN) [24] is currently the most mature and representative deep learning algorithm, particularly excelling at processing data, especially images. CNN creatively proposed an input layer, multiple hidden layers, and an output layer, where the hidden layer includes components such as the convolution layer, the pooling layer, and the fully connected layer. The core strength of CNN lies in its ability to automatically extract features from input data through the convolution layer, while the pooling layer reduces the size of the data space, and the fully connected layer facilitates classification tasks. Of course, it is also suitable for regression tasks such as temperature prediction. The CNN model structure layer is shown in Figure 10.

The convolution layer is the core component of CNN. Its main function is to perform a convolution operation on the input grain storage temperature data and then output it to the next layer of the network. The convolution operation is defined as follows:

(11) $$f_{t}=\sigma (W_{xf}x_{t}+W_{hf}h_{t-1}+b_{f})$$

$h_{li}$ is the i−th feature of the l−th layer l is the activation function, $W_{li}$ is the i−th convolution kernel weigh matrix of the l−th layer, operator ∗ is the convolution operation, $x_{l-1}$ is the output of (l−1)−th layer, and $b_{li}$ is the bias term.

After the grain storage temperature data are processed by convolution, an activation function is applied to capture the nonlinear characteristics of the output data and enhance the feature representation ability of the model. Traditionally, sigmoid and tanh functions were initially used as activation functions in CNN. However, the ReLU function is now widely used in CNN models due to its advantages such as faster convergence and a simple gradient. The expression of the ReLU function is as follows:

(12) $$f_{cov}(h_{i}^{l})=\max(0,h_{i}^{l})$$

To ensure that sufficient feature vectors are extracted, CNN often requires a large number of parameters. The introduction of the pooling layer effectively addresses this issue by retaining the most important features of the input data. Commonly used pooling layers include average pooling and maximum pooling. In this article, the maximum pooling is used:

(13) $$y_{i}^{l+1}(j)=\underset{k\in D_{j}}{\max}\{x_{i}^{l}(k)\}$$

$y_{i}^{l+1}(j)$ is the element in the i−th feature of the (l+1)−th layer after max pooling, $x_{i}^{l}(k)$ is the element in the i−th feature of the l−th layer in the pooling kernel, and $D_{j}$ is the j−th pooling region.

The Fully Connected (FC) module is the final module of the CNN model, which uses the extracted nonlinear activation features to generate the probability distribution of each classification and outputs the model using the Softmax function.

(14) $$p(y_{j})=\frac{\exp(y_{j})}{\sum_{k=1}^{m}\exp(y_{k})}$$

$y_{j}$ is the output of the j−th neuron in the output layer, m is the number of classification task categories, and $p(y_{j})$ is the probability output of the neuron after passing through the Softmax fuction.

#### 2.3.3. SENet (Squeeze-And-Excitation Net)

**Figure 11:**
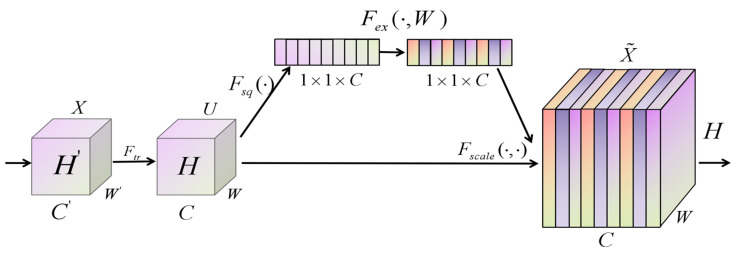
The structure of the SENet module.

In 2017, Hu Jie et al. [25] proposed a channel attention mechanism: the SENet (Squeeze-and-Excitation Net) attention mechanism. This unit architecture explicitly establishes the interdependence between channels and adaptively recalibrates channel features, allowing the neural network to pay more attention to the important features of the current task and suppress less useful features. The structure of the SENet module is shown in Figure 11.

In the SENet module, the two most important components are the Squeeze and Excitation operations. First, feature C undergoes the Squeeze operation, which generates a channel descriptor by aggregating feature maps (H×W) across spatial dimensions, capturing the global distribution of channel features. Then the Excitation operation is performed, using a gating mechanism consisting of a full connection and sigmoid activation function to learn the importance of multiple channels in the task. Finally, it assigns higher weights to important features while suppressing and reducing less important ones.

(15) $$z_{c}=F_{sq}(u_{c})=\frac{1}{H\times W}\sum_{i=1}^{H}\sum_{j=1}^{W}u_{c}(i,j)$$

(16) $$s=F_{ex}(z,W)=\sigma (g(z,W))=\sigma (W_{2}\delta (W_{1}z))$$

(17) $${\tilde{x}}_{c}=F_{scale}(u_{c},s_{c})=s_{c}u_{c}$$

Among them, $u_{c}$ and $z_{c}$ represent the output mapping of the c−th channel after convolution transformation and global feature compression transformation, respectively, σ represents the sigmoid function, $W_{1}$ and $W_{2}$ represent the weight matrices of the two fully connected layers, respectively, and ${\tilde{x}}_{c}$ represents the output matrix of the c−th channel after weight calibration. The final output matrix of the l−th layer network in a single structure block of SE-DenseNet is $X=[x_{1},x_{2},x_{3},\ldots ,x_{n}]$.

#### 2.3.4. Comparative Analysis of Selected Models

The selected models were compared and analyzed, and the analysis results are shown in Table 1.

#### 2.3.5. FTA-CNN-SE-LSTM

**Figure 12:**
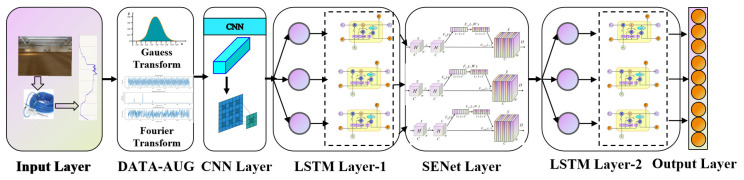
FTA-CNN-SE-LSTM model structure.

The FTA-CNN-SE-LSTM grain storage temperature prediction model proposed in this paper includes the following: a dual-domain data augmentation module in the time domain and frequency domain based on Gaussian noise and fast Fourier transform (FFT), a CNN layer, the SENet channel attention mechanism, two LSTM layers, and a fully connected layer. The model structure diagram is shown in Figure 6. In the first layer, Gaussian noise and FFT are applied to augment the data in the time domain and frequency domain for less grain storage temperature data, so as to avoid the data quality and quantity problems caused by the small amount of data or sensor collection errors. As a result, the characteristics of the grain storage temperature data input to the model become more obvious, and small changes are more prominent, which lays a good foundation for the subsequent model feature learning. Then, relying on the powerful feature extraction ability of the convolution kernel in the CNN convolution layer, the most significant features of the grain storage temperature are extracted. The maximum pooling layer is used to upsample and downsample the convolutional layer output data in the time dimension. In the second layer, the SENet module is added to adjust the grain storage temperature feature vector extracted in the convolution layer, and the channel weight is corrected. The important features of the grain storage temperature are highlighted, and the inconspicuous features are suppressed. In the third layer, the features processed by the SENet module are input into the LSTM layers to extract the temporal dependent information of the grain storage temperature data, and then the grain storage temperature features are further extracted. Dropout and L2 regularization are used to prevent the model from overfitting. After two layers of LSTM-based feature extraction, the feature vector is input into the fully connected layer to predict the grain storage temperature, and finally the temperature prediction result of the grain storage temperature prediction model is output. The model structure is shown in Figure 12. The model structure parameters are shown in Table 2.

### 2.4. Standardization of Grain Storage Temperature Data

Before the grain storage temperature data are input into the prediction model, it is necessary to normalize the data to the same level. After data normalization [26], the prediction model will learn the optimal solution faster during the training process, improve the convergence speed, and avoid the problem of excessive model prediction error caused by data level problems. The data normalization method used in this paper is Z-score normalization [27], and the calculation formula is as follows:

(18) $$x^{\prime }=\frac{x-\mu }{\sigma }$$

Among them, x represents the grain storage temperature data, μ and σ represent the mean and standard deviation of the grain storage temperature sample data, respectively, and $x^{\prime }$ represents the normalized result of the grain storage temperature. After normalization, the grain storage temperature data present a standard normal distribution. When the prediction results are finally output, the actual predicted temperature data can be obtained by applying the inverse normalization process.

### 2.5. Prediction Evaluation Indicators

To evaluate the performance of the grain storage temperature prediction model in actual prediction and to reflect the performance improvement of the model after each improvement and optimization, the mean absolute error (MAE) and root mean square error (RMSE) were used as evaluation metrics of the model prediction results.

MAE [28] is a common loss function in regression tasks. It calculates the average of the sum of the absolute values of the differences between the predicted values and the actual values. The smaller the value, the closer the model-predicted grain storage temperature is to the actual grain storage temperature. m represents the number of output samples, $x_{i}$ represents the actual grain temperature value, and ${\hat{x}}_{i}$ represents the predicted grain temperature value.

(19) $$MAE(x,\hat{x})=\frac{1}{m}\sum_{i=1}^{m}\left(|x_{i}-{\hat{x}}_{i}|\right)$$

RMSE [29] is the square root of the mean square error (MSE) [30], which is calculated as the square root of the sum of the squares of the differences between the predicted value and the true value of the stored grain temperature. The smaller the value, the smaller the model prediction error and the stronger the model prediction ability.

(20) $$RMSE(x,\hat{x})=\sqrt{\frac{1}{m}\sum_{i=1}^{m}{\left(x_{i}-{\hat{x}}_{i}\right)}^{2}}$$

### 2.6. Dataset Partitioning

In this experiment to determine the basic model, when dividing the data set, 80% of the original grain storage temperature data were used as the training set, and the remaining 20% of the grain storage temperature data were used as the test set.

### 2.7. Prediction Setting

In the prediction section of this study, we use the grain storage temperature data from the past ten days as input to perform a one-step prediction, specifically forecasting the temperature for the following day. Given that grain storage temperature exhibits short-term coherence and predictability, this setup enables the model to focus on identifying patterns and characteristics associated with the temperature change for the following day based on the past ten days’ data, resulting in more accurate predictions.

## 3. Results and Discussion

### 3.1. Comparison and Selection of Basic Models

In order to prove that the LSTM network has the best performance as the basic network of this experiment, this experiment will build grain storage temperature prediction models based on the least squares method (LSM) [31], BP neural network [32], convolutional neural network (CNN) [33], gated unit [34], causal convolution [35], transformer [36], LSTM, and other networks and compare their prediction performance.

**Figure 13:**
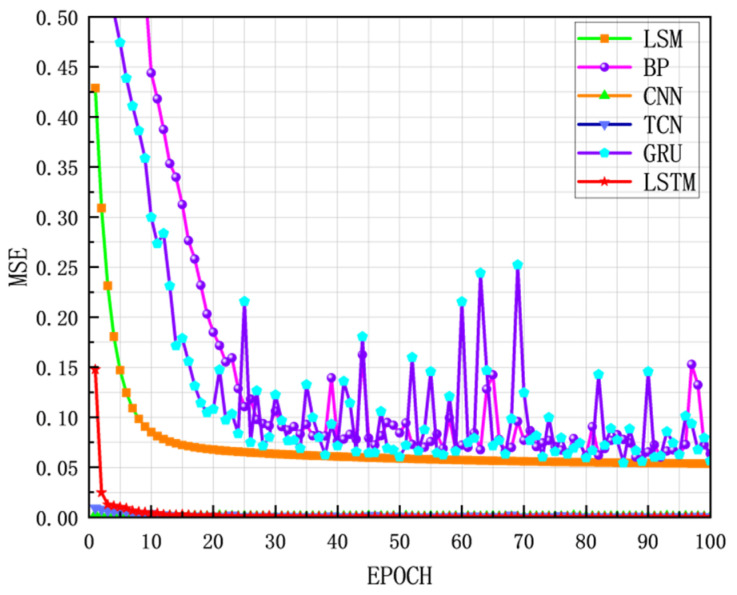
Comparison of training loss curves of different basic models.

**Figure 14:**
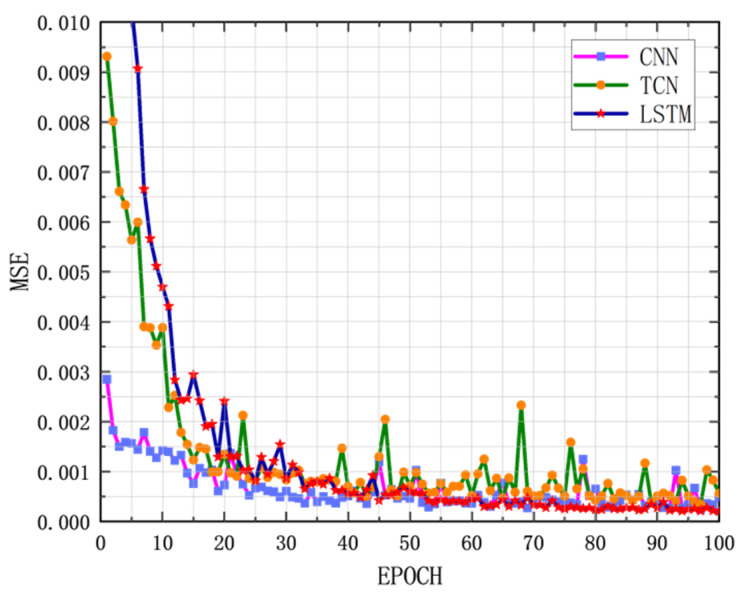
Comparison and enlargement of the best basic model training loss curve.

Through 100 rounds of experiments, it was concluded that the grain storage temperature was predicted using the traditional mathematical method, LSM, the MAE of the model prediction was 0.1001, and the RMSE was 0.2513. A BP neural network model was constructed, which was divided into three layers. The first layer was the input layer, with 64 neurons and ReLU as the activation function. The second layer was the hidden layer, with 32 neurons and ReLU as the activation function. The third layer was the output layer. The BP neural network grain storage temperature prediction model was obtained through the experiment. The MAE of the prediction was 0.0553, and the RMSE was 0.0681. Compared with the traditional mathematical model, the MAE was reduced by 44.8%, and the RMSE was reduced by 72.9%, indicating that the deep learning method is more suitable for the task of grain storage temperature prediction. The gated unit GRU model is constructed. The core layer is the GRU layer, which has 64 neurons in total. ReLU is used as the activation function. The MAE of the grain storage temperature prediction model based on GRU is 0.0474, and the RMSE is 0.0585. Compared with the BP neural network, the MAE is reduced by 44.73%, and the RMSE is reduced by 14.14%, indicating that GRU has high applicability and good performance for time series prediction tasks. As the most commonly used model for image processing in deep learning, the CNN can also be applied to time series prediction tasks due to its excellent feature extraction ability of the convolutional layer. The prediction of grain storage temperature is realized by the components of convolutional layer, pooling layer, and fully connected layer. The MAE of the prediction is 0.0348, and the RMSE is 0.0396. Compared with the BP neural network, the prediction performance has been greatly improved: the MAE is reduced by 26.80%, and the RMSE is reduced by 32.98%. The temporal convolutional network (TCN) is a time series prediction task model that has emerged in recent years. To build a TCN model, the first layer uses causal convolution, the second layer uses ordinary convolution and a pooling layer, the third layer uses a convolution layer and a pooling layer, and the model finally uses a flattening layer and a fully connected layer to build a prediction model. The MAE of the prediction is 0.0291, and the RMSE is 0.0329. Compared with CNN, its MAE is reduced by 16.36%, the RMSE is reduced by 16.92%, and the performance is good. The transformer is one of the main models for time series prediction tasks. In this experiment, the MAE of the prediction is 10.2899, and the RMSE is 12.0027. The prediction effect is very poor, indicating that the transformer model has difficulty reflecting its advantages in processing tasks for small sample time series prediction tasks. A simple long short-term memory network (LSTM) is constructed. A total of eight neurons are built in the LSTM layer in the model to accept the input of time series data. Then a single neuron in the dense layer is built. The output of the generative model is used to complete the regression task. The LSTM grain storage temperature prediction model achieved an MAE of 0.0212 and an RMSE of 0.0262. Its prediction performance is better than that of TCN, with the MAE reduction of 27.14% and the RMSE reduction of 20.39%. Compared with the initial model LSV, the MAE is reduced by 78.79%, and the RMSE is reduced by 89.54%. It can be clearly concluded that LSTM has shown the best prediction performance in the task of predicting grain storage temperature based on the current small sample grain data. The prediction performance comparison is shown in Table 3, and the prediction performance loss curve is shown in Figure 13. The loss curves of TCN, CNN, and LSTM with excellent prediction performance are presented separately in Figure 14.

### 3.2. Improved Model Predictive Performance

When LSTM is determined as the basis of the model, the input layer of the model is replaced with the CNN convolution layer. Relying on the powerful feature extraction ability of the convolution layer and combining it with the LSTM model’s own learning advantage in the time dependency relationship of time series data, the model’s ability to understand and capture the characteristics of time series data is further improved. In this configuration, the CNN-LSTM model achieved an MAE of 0.0138 and an RMSE of 0.0213 in the prediction task. Compared with the basic model LSTM, the MAE and RMSE are reduced by 34.91% and 18.83%, respectively, which can better realize the prediction of stored grain temperature. To further improve the performance of the prediction model to the extreme at the level of model structure improvement, the SENet (Squeeze-and-Excitation Networks) attention module is introduced to enable the model to pay more attention to the characteristics of grain storage temperature data under limited data conditions. It highlights the features that are important for model learning, suppresses the features that are not important for model learning, controls the feature vectors input into the LSTM layer, and improves the learning content quality of the prediction model. After the introduction of the SENet attention module, the MAE of the CNN-SE-LSTM grain storage temperature prediction model has reached 0.0123, and the RMSE has reached 0.0147. Compared with the CNN-LSTM model, the performance has been significantly improved, with the MAE and RMSE reduced by 10.87% and 30.56%, respectively.

Through the results of multiple rounds of experiments, it can be concluded that the optimal structure of the grain storage temperature prediction model has been achieved at the model level. However, at present, the actual application scenarios of the prediction model and the data collected in this experiment are mostly troubled by the amount of data and the data quality. This issue in turn affects the source of high-quality learning data for the deep learning model and ultimately leads to poor prediction performance of the grain storage temperature prediction model, making it difficult to cope with the temperature prediction task of the actual granary. Data serve as the foundation for deep learning models. On the basis of existing data, to address this issue, it is proposed to augment the grain storage temperature data through the two dimensions of frequency domain and time domain. By adding Gaussian noise to the grain storage temperature data and using FFT to expand the grain storage temperature data features from the time domain dimension to the frequency domain dimension, this approach makes the features in the model’s input data more prominent, enhancing both time-domain and frequency-domain features of the grain storage temperature data. The data quality of the input model is improved without changing the original data, so that the model can learn more important features that are difficult to find. To this end, after completing the improvement of the model structure, the training and validation sets were readjusted, and 80% of the original grain storage temperature data was used as the training set after time domain and frequency domain augmentation, and the last 20% of the original unaugmented data was used as the testing set to facilitate subsequent experiments.

**Figure 15:**
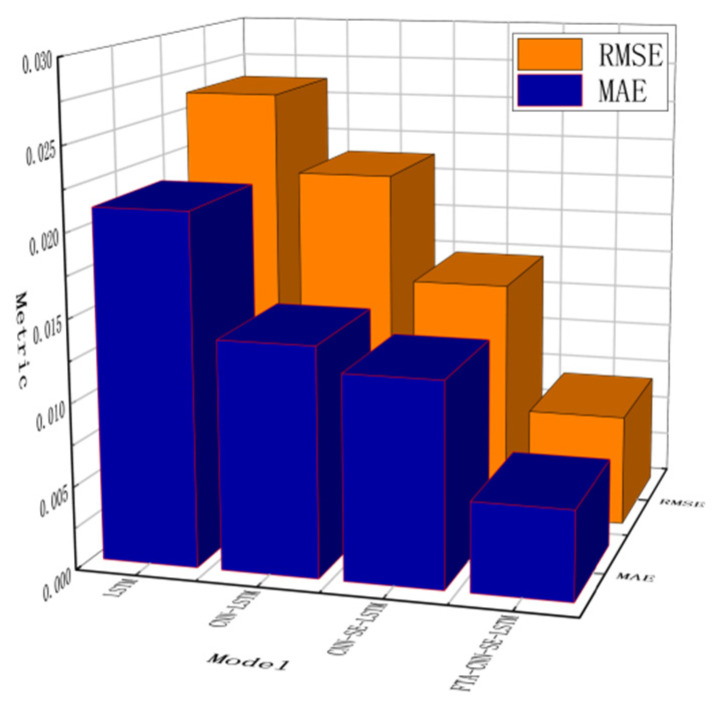
Three-dimensional display of the improvement effect of the LSTM model after improvement.

**Figure 16:**
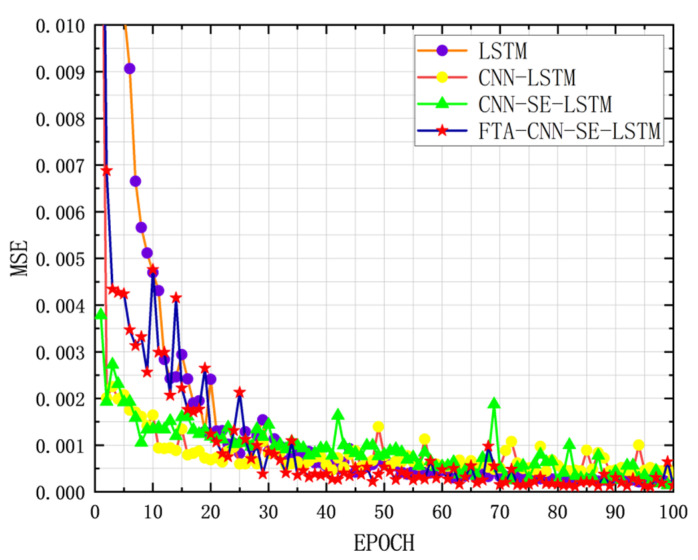
Comparison of training loss after LSTM model improvement.

After introducing data augmentation, the FTA-CNN-SE-LSTM grain storage temperature prediction model achieved an MAE of 0.0054 and an RMSE of 0.0068. Compared with the original CNN-SE-LSTM model, the MAE and RMSE are reduced by 56.10% and 56.06%, respectively. Compared with the original LSTM model, the MAE and RMSE are reduced by 74.77% and 74.02%, respectively. By enhancing the learning content quality at the data level, the model’s prediction performance improved significantly, reaching its best prediction level. The performance comparison of each model in the improvement process is shown in Table 4, the three-dimensional display of the improvement effect is shown in Figure 15, and the loss curve is presented in Figure 16.

In order to conduct a more comprehensive evaluation of the performance of the FTA-CNN-SE-LSTM grain storage temperature prediction model proposed in this paper, studies similar to it in the field of grain storage temperature prediction were selected for comparison. Sun et al. [37] proposed the C-ERBF model, which combines chaos theory with the enhanced radial basis neural network to predict grain storage temperature. Using this model to conduct experiments on the data of this paper, the results showed that the MAE was 0.0132 and the RMSE was 0.0114. Zhu et al. [38] proposed the TCN-BiGRU model, which extracts the features of grain storage temperature through TCN and introduces the attention mechanism to enhance the accuracy of predicting grain storage temperature by feature enhancement. Using this model to conduct experiments on the data of this paper, the results showed that the MAE was 0.0919 and the RMSE was 0.1082. Using the GA-PSO-GRU-Attention model proposed by Liu et al., experiments were conducted on the data of this paper, and the results showed that the MAE was 0.0460 and the RMSE was 0.0547. Through the above experimental comparison, it can be concluded that the FTA-CNN-SE-LSTM model proposed in this paper has better prediction performance in grain storage temperature prediction.

**Figure 17:**
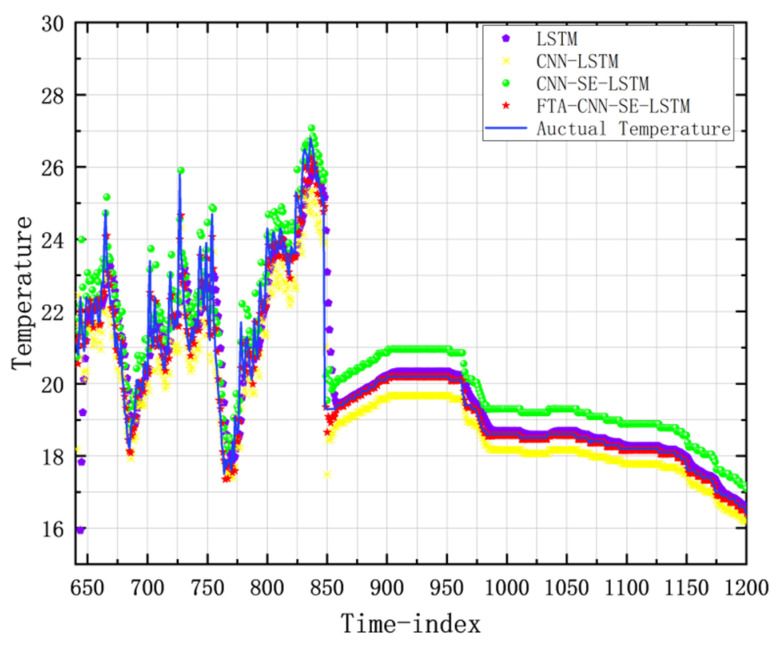
Comparison of the LSTM model improved predicted temperature points and the actual grain storage temperature curve.

To evaluate the performance of the model in the temperature prediction task of the actual granary, in addition to observing and comparing the training indicators, the actual data collected in the granary should be used for actual prediction. In this experiment, the 0–1200 segment of a large dataset of grain storage temperature was used for prediction. The results are depicted in the point-line graph shown in Figure 17. As can be observed, the prediction points of FTA-CNN-SE-LSTM can best fit the actual grain storage temperature data curve. The real and predicted values of 10 randomly selected grain storage temperatures are displayed in Table 5. These results confirm that the FTA-CNN-SE-LSTM model achieves the best performance in predicting actual grain storage temperatures.

In the above experiments, the CNN-SE-LSTM grain storage temperature prediction model proposed in this paper showed the best prediction performance in the prediction task compared with other models in terms of model structure, and the performance of the FTA-CNN-SE-LSTM grain storage temperature prediction model was further improved after the introduction of time domain and frequency domain data augmentation. As a result, the FTA-CNN-SE-LSTM grain storage temperature prediction model achieved the best performance in terms of both prediction accuracy and real-world grain storage temperature prediction tasks.

## 4. Conclusions

In view of the complexity of the actual grain storage environment inside the granary, this paper first proposed a CNN-SE-LSTM grain storage temperature prediction model based on deep learning. The basic network LSTM of this prediction model was selected through comparative experiments with other similar networks and is more suitable for the task of grain storage temperature prediction. The convolution layer and the SENet (Squeeze-and-Excitation Networks) attention module were introduced. This model gives full play to the CNN’s ability to extract features and the LSTM’s ability to capture the time dependency of time series data. The SENet attention module highlights the important features of the model and suppresses unnecessary features. Through the above methods, the prediction performance of this model has been significantly improved in terms of structure. Compared with the basic model LSTM, the MAE has been reduced by 74.77%, and the RMSE has been reduced by 74.02%. However, one thing that cannot be ignored is that in the actual management of grain storage in granaries, due to the particularity and confidentiality of grain storage units, the amount of grain storage temperature data is very limited, and there are uncontrollable abnormalities in grain condition measurement and control, resulting in problems with data quality and quantity. This is also the reason why the improvement of the model’s prediction performance has encountered bottlenecks despite the structural changes. In view of the small sample size and the difficult-to-control data quality, we augment the grain storage temperature data in both the time domain and frequency domain. Specifically, we add Gaussian noise to the data in the time domain dimension to improve the model’s ability to discover and learn subtle changes in time series data, and we apply FFT to expand the data from the time domain to the frequency domain, enabling the model to capture the periodicity of time series data in two dimensions. Through data augmentation processing, the bottleneck problem of prediction performance after structural improvement of the model due to small sample data is well solved. Compared with the best model CNN-SE-LSTM after model structure improvement, the FTA-CNN-SE-LSTM model after data augmentation achieves a 56.10% reduction in MAE and a 56.06% reduction in RMSE. The FTA-CNN-SE-LSTM model proposed in this paper achieves the best performance in the grain storage temperature prediction task under the actual challenge of small sample data. Compared with the current similar research in the field of grain storage temperature prediction, the model proposed in this paper has excellent performance, and the method is more innovative, which is beneficial to promote the development of grain storage temperature prediction technology.

In the actual research process, the prediction method of grain storage temperature can be further explored in many aspects. For example, in this experiment, only the average temperature of a certain layer of the entire warehouse was considered as the input factor of the model, that is, a single variable prediction was performed. However, in the actual granary, more types of sensor equipment can be deployed to collect different types of grain storage environment data, such as granary temperature, atmospheric temperature, granary humidity, atmospheric humidity, and other factors. There is a certain influential relationship between these grain storage environment factors, which can provide more abundant grain storage information. Using these factors for multivariate prediction can make the prediction results more comprehensive and accurate.

## Figures and Tables

**Table 1:** Comparative analysis of selected models.

Model	Features	Advantage	Limitation
LSTM	It has memory units and gating mechanisms that can transmit information in time series and control the inflow, outflow, and retention of information. It can capture long-term and short-term dependencies.	It can effectively handle long-term dependencies in grain storage temperature data, such as capturing seasonal temperature change trends. It has good modeling capabilities for time series patterns of temperature fluctuations and adapts to the complex changes in grain storage temperature.	The computational complexity is high, and the training takes a long time when processing a large amount of grain storage temperature data. There are many parameters, and it is easy to overfit when the amount of data is limited, which affects the prediction accuracy.
CNN	The convolution layer extracts local features by sliding the convolution kernel and sharing parameters. The pooling layer downsamples the feature map to reduce the data dimension and builds a multi-layer hierarchy for feature extraction.	It can extract local features of grain storage temperature data, such as the temperature change pattern at a specific time of day. It reduces the number of parameters and the amount of calculation, and has relatively high training efficiency, making it suitable for processing large-scale grain storage temperature data.	It is difficult to directly process long-sequence time dependencies. The temperature of stored grain is affected by many factors over a long period of time, so it performs poorly in this aspect. It does not fully utilize the time sequence information of stored grain temperature data as LSTM does.
SENet	The Squeeze and Excitation modules are introduced to capture inter-channel dependencies through global average pooling. They adaptively reweight channel features to emphasize important feature channels.	In the prediction of stored grain temperature, feature channels closely related to temperature changes can be highlighted, such as the importance adjustment of sensor data at different locations. The structure is lightweight and can be embedded in other models (such as LSTM, CNN) to improve prediction performance with little increase in computational overhead.	When used alone, it is not very targeted for the task of predicting stored grain temperature. It may overemphasize certain channel features and ignore other potentially important information.

**Table 2:** Model structure parameters.

Structure Name	Number of Channels	Convolution Kernel Size	Hidden Layer Size	Activation Function
Convolutional Layer	64	3	--	ReLU
Max Pooling Layer	64	2	--	
LSTM Layer-1	--	--	64	tanh
SENet	--	--	--	ReLU, sigmoid
LSTM Layer-2	--	--	32	tanh

**Table 3:** Comparison of various indicators between LSTM network and basic networks such as BP, GRU, transformer, TCN, and CNN.

Model	MAE	RMSE
LSM	0.1001	0.2513
BP	0.0553	0.0681
GRU	0.0474	0.0585
CNN	0.0348	0.0396
TCN	0.0291	0.0329
Transformer	10.2899	12.0027
LSTM	0.0212	0.0262

**Table 4:** Comparison of various indicators after LSTM network improvement.

Model	MAE	RMSE
LSTM	0.0212	0.0262
CNN-LSTM	0.0138	0.0213
CNN-SE-LSTM	0.0123	0.0147
FTA-CNN-SE-LSTM	0.0054	0.0068

**Table 5:** Comparison between the actual value of random points of grain storage temperature and the model prediction value.

Number	Actual Temperature	Predicted Temperature
1	−11.50	−11.49
2	−9.50	−9.52
3	−4.30	−4.33
4	3.90	3.90
5	21.70	21.68
6	20.10	20.10
7	18.60	18.61
8	15.90	15.87
9	7.80	7.79
10	−0.10	−0.10

## Data Availability

The original contributions presented in this study are included in the article. Further inquiries can be directed to the corresponding author.
